# Simultaneous Optimization of the Production of Organic Selenium and Cell Biomass in *Saccharomyces Cerevisiae* by Plackett-Burman and Box-Behnken Design

**Published:** 2018

**Authors:** Hamed Zare, Parviz Owlia, Hossein Vahidi, Maryam Hosseindokht Khujin

**Affiliations:** a *Department* *of* *Pharmaceutical Biotechnology, School of Pharmacy, Shahid Beheshti University of Medical Sciences, Tehran, Iran. *; b *Student Research Committee, Shahid Beheshti University of Medical Sciences, Tehran, Iran.*; c *Molecular Microbiology Research Center, Faculty of Medicine, Shahed University, Tehran, Iran.*; d *Department of Genetics and Molecular Biology, School of Medicine, Isfahan University of Medical Sciences, Isfahan, Iran.*

**Keywords:** Selenium, Yeast, Plackett-Burman, Box-Behnken, Optimization

## Abstract

Selenium (Se) as a vital trace element has many biological activities such as anti-inflammation and anti-oxidation. Selenomethionine as an organic selenium plays a vital role in the response to oxidative stress. At present, *Saccharomyces cerevisiae* is one of the best microorganisms that has the ability to accumulate selenium. Production of Seleno-yeast was done by growing *Saccharomyces cerevisiae* in the presence of water soluble selenium salt (Na_2_SeO_3_) as a part of the medium. The yield of selenium biotransformation and yeast biomass can be improved by optimizing the process conditions in two steps. First, the effects of several culture parameters (culture conditions and culture media) were studied using the Plackett-Burman design. After that, determining the optimum levels of the effective parameters was performed by Box-Behnken response surface methodology. Optimization of the conditions was performed with the aim of simultaneously optimizing the biomass and selenium biotransformation. In this investigation, the effect of the eleven culture parameters was studied with Plackett-Burman design. Then, four significant culture parameters such as glucose concentration, aeration, selenium concentration, and temperature were optimized with Box-Behnken response surface methodology.

## Introduction

Selenium (Se) as a vital trace element for the growth of mammals was discovered by Berzelius in 1817 (1-4). This element incorporates numerous enzymes and selenoproteins and has many biological activities such as anti-inflammation and anti-oxidation (2). Selenium deficiency causes some problems, which include: arthritis, infertility, and Keshan disease in humans (3, 5). Also, some studies have shown that selenium reduces free radicals and delays the development of some of the chronic diseases, such as cancer and heart disease (4, 6-8). As a result, food supplementation by selenium is considered to prevent selenium deficiency (3). On the other hand, ingestion of organic selenium compounds and selenium-containing amino acids are safer and better than the inorganic selenium (1, 9). Studies have shown some microorganisms, especially yeast and lactic bacteria, can accumulate great amounts of inorganic selenium and transform into organic form, mainly selenomethionine (9-11). Selenomethionine is safe, highly bioactive and is the best source of selenium for organisms (4, 11). Production of Seleno-yeast was done by growing *Saccharomyces cerevisiae* in the presence of water soluble selenium salt (Na_2_SeO_3_) as a part of the medium (12). Seleno-yeast production is affected by various parameters including pH, dissolved oxygen, temperature, incubation time, agitation rate and volume of inoculums (13). In addition, several media component such as yeast extract, selenium concentration and peptone are the parameters which influence on the selenium incorporation into yeast cells (2, 3 and 14). Consequently, the optimizations of the major factors are essential to find their optimum levels. 

The yield of selenium biotransformation and yeast biomass can be improved by optimizing the process conditions (13, 15). The usual method for optimization involves changing one of the factors at a time, and keeping other factors constant (15, 16). This approach is easy and simple to implementation, but it may be very time consuming and costly (15). Response surface methodology (RSM) is a group of statistical and mathematical methods used for experiment designing (11). Reducing the number of experimental trials is the main advantage of RSM technique (9). A helpful method of RSM for optimizing processes is Box-Behnken that is widely used in experimental designing (11). In order to decrease the number of experimental trials, it is advisable to screen the factors before optimization and find the effective factors (17). The Plackett-Burman design (PBD) is especially helpful for screening the parameters that has the most effect on a reaction (17). This technique can estimate the major effects of N parameters only in N + 1 experiment (16).

In this investigation, screening for approximation of importance of eleven process parameters including culture conditions and medium ingredients on selenium biotransformation and Seleno-yeast biomass production was investigated by PBD. In the following, to find the best conditions for the Se biotransformation in yeast cells, optimization of impact factors were performed by Box-

Behnken.

## Experimental

*Materials and microorganism*

The media component such as dextrose, peptone, NaCl and the other chemicals were purchased from Merck Co. Darmstadt, Germany. Na_2_SeO_3_ prepared from Merck Co. of analytical grade. The yeast isolate (*Saccharomyces cerevisiae*) was obtained from our previous study (4). This strain was selected by resistance screening with a concentration of 25 mg/L of sodium selenite Na_2_SeO_3_ and was stored in Sabouraud dextrose (SD) broth.

*Determination of organic selenium production (selenium biotransformation)*

The yeast culture broth was centrifuged at 8000×g for 10 min. For the removal of unbound selenium, the yeast cell pellets were washed three times by deionized water and then centrifuged (8000×g for 10 min). The yeast cells were dried at 85 °C to reach a constant weight. Determination of selenium content was carried out according to the atomic absorption spectroscopy (AAS) method (17). About 0.2 g of the dried yeast cell was digested with 10 mL of concentrated HNO_3_ (65%) for 15 min at 105 °C in a digestive flask. Afterward, the solution was cooled and in order to reducing Se (‏^6^) to Se (‏^4^) and also completing the digestion procedure, the solution was heated with 2 mL of concentrated HCl (37%) for 10 min at 80 °C. A reflux device was applied during digestion process, to prevent from volatilization of selenium. 

This solution was cooled and a constant volume was created (by ultra-pure water) and used for total selenium determination by AAS. It is notable that the blank was prepared in the same way. For inorganic selenium measurement, 0.2 g of the dried yeast mixed with ultra-pure water was heated in boiling bath for 1 h. Next, the mixture was centrifuged at 8000×g for 15 min. Then the supernatant was filtrated and a constant volume was made (by ultra-pure water) and used for inorganic selenium measurement by AAS. Calculation of organic selenium (selenium biotransformation) was attained from the difference among the total selenium and inorganic selenium yield (11).

**Table 1:** Values for Plackett-Burman design

**Low level**	**High level**	**Variable**	**Factor**
5 mg/L	25 mg/L	A	Selenium concentration
25 °C	31 °C	B	Temperature
24 h	48 h	C	Inoculum age
15 g/L	25 g/L	D	Glucose concentration
4	7	E	pH
150 rpm	250 rpm	F	Aeration
1%	3%	G	Inoculum volume
50 mL	100 mL	H	Volume
0 h	9 h	I	Selenium adding time
24 h	72 h	J	Incubation time
7 g/L	13 g/L	K	Peptone concentration

**Table 2:** Placket-Burman design table with organic selenium (selenium biotransformation) results. A-K are screening parameters and trails No. 13 to 17 are center points. The amount obtained for organic selenium is derived from experimental data.

**Trial No.**		**Vaiables**											**Organic selenium**
		**A**	**B**	**C**	**D**	**E**	**F**	**G**	**H**	**I**	**J**	**K**	
	**Unit**	**mg/L**	**Cº**	**h**	**g/L**	**pH**	**rpm**	**%**	**mL**	**H**	**h**	**g/L**	**(ppm)**
1		25	31	24	25	7	250	1	50	0	72	7	1350
2		5	31	48	15	7	250	3	50	0	24	13	1580
3		25	25	48	25	4	250	3	100	0	24	7	2850
4		5	31	24	25	7	150	3	100	9	24	7	1425
5		5	25	48	15	7	250	1	100	9	72	7	1175
6		5	25	24	25	4	250	3	50	9	72	13	1419
7		25	25	24	15	7	150	3	100	0	72	13	1291
8		25	31	24	15	4	250	1	100	9	24	13	1021
9		25	31	48	15	4	150	3	50	9	72	7	1740
10		5	31	48	25	4	150	1	100	0	72	13	517
11		25	25	48	25	7	150	1	50	9	24	13	1363
12		5	25	24	15	4	150	1	50	9	24	7	658
13		15	28	36	20	5	200	2	75	4.5	48	10	1457
14		15	28	36	20	5	200	2	75	4.5	48	10	1504
15		15	28	36	20	5	200	2	75	4.5	48	10	1551
16		15	28	36	20	5	200	2	75	4.5	48	10	1598
17		15	28	36	20	5	200	2	75	4.5	48	10	1645

**Table 3:** Coded and real values for the Box-Behnken design

**Factor**	**Variable**			Level of variable
		**-1**	**0**	**+1**
Selenium concentration	A	5 mg/L	15	25 mg/L
Temperature	B	25 °C	28	31 °C
Glucose concentration	D	15 g/L	20	25 g/L
Aeration	F	150 rpm	200	250 rpm

**Table 4:** Box-Behnken design table; (+1): high level; (–1): low level; (0) central point. The amount obtained for biomass and organic selenium is derived from experimental data.

**Trial No.**	**Variables**				**Biomass (g/L)**	**Organic selenium (ppm)**
	**D** **(Glucose)**	**F** **(Aeration)**	**A** **(Selenium)**	**B** **(Temperature)**		
1	-1	-1	0	0	1.16	1734
2	1	-1	0	0	3.67	1632
3	-1	1	0	0	3.64	1967
4	1	1	0	0	6.23	1957
5	0	0	-1	-1	3.99	506
6	0	0	1	-1	4.18	1363
7	0	0	-1	1	4.29	1705
8	0	0	1	1	4.27	2609
9	-1	0	0	-1	3.25	1249
10	1	0	0	-1	4.94	1202
11	-1	0	0	1	1.89	2429
12	1	0	0	1	4.59	2482
13	0	-1	-1	0	3.29	980
14	0	1	-1	0	5.59	1320
15	0	-1	1	0	2.76	1789
16	0	1	1	0	5.25	2147
17	-1	0	-1	0	2.99	1139
18	1	0	-1	0	4.45	1054
19	-1	0	1	0	2.43	1934
20	1	0	1	0	4.78	1987
21	0	-1	0	-1	2.80	1110
22	0	1	0	-1	5.83	1318
23	0	-1	0	1	2.61	2334
24	0	1	0	1	5.73	2535
25	0	0	0	0	4.18	1857
26	0	0	0	0	4.38	1876
27	0	0	0	0	4.41	1916
28	0	0	0	0	4.45	1934
29	0	0	0	0	4.54	1897

**Table 5:** ANOVA for Plackett-Burman design (selenium biotransformation response).

**Source**	**Sum of Squares**	**df**	**Mean Square**	***f*** **-value**	***p*** **-value** **(Prob ˃ F)**
Model	7.94 E+006	4	1.98 E+006	61.7	˂0.0001
Selenium concentration	5.49 E+006	1	5.49 E+006	132.44	˂0.0001
Temperature	1.79 E+006	1	1.79 E+006	41.05	˂0.0001
Glucose concentration	3.70 E+005	1	3.70 E+005	6.33	0.0167
Fermentation time	2.90 E+005	1	2.90 E+005	8.53	0.0281
Curvature	7.30 E+005	1	7.30 E+005	13.44	0.0017
Residual	4.89 E+005	11	44427.78		
Lack of fit	4.40 E+005	7	62970.05	6.25	0.0690
Pure Error	47915.20	4	11978.80		
Cor Total	9.16 E+006	16			

**Table 6:** ANOVA for Plackett-Burman design (yeast biomass response).

**Source**	**Sum of Squares**	**df**	**Mean Square**	***f*** **-value**	***p*** **-value** **(Prob ˃ F)**
Model	42.11	4	10.53	44.73	˂0.0001
Glucose concentration	29.11	1	29.11	123.68	˂0.0001
Aeration rate	9.49	1	9.49	40.31	˂0.0001
Temperature	1.96	1	1.96	8.33	0.0148
Selenium concentration	1.55	1	1.55	6.58	0.0263
Curvature	3.86	1	3.86	16.38	0.0019
Residual	2.59	11	0.24		
Lack of fit	2.33	7	0.33	5.23	0.0646
Pure Error	0.26	4	0.064		
Cor Total	48.55	16			

**Table 7:** ANOVA for response surface quadratic model (Box-Behnken for selenium biotransformation).

**Source**	**Sum of squares**	**df**	**Mean Square**	***f*** **-value**	***p*** **-value** **(Prob ˃ F)**
Model	7.59 E+006	14	5.43 E+005	371.63	˂0.0001
D- Glucose	1587	1	1587	1.09	0.3148
F- Aeration	2.31 E+005	1	2.31 E+005	158.23	˂0.0001
A- Selenium	2.19 E+006	1	2.19 E+006	1499.17	˂0.0001
B- Temperature	4.49 E+006	1	4.49 E+006	3080.11	˂0.0001
DF	2116	1	2116	1.45	0.2486
DA	4761	1	4761	3.26	0.0925
DB	2500	1	2500	1.71	0.2118
FA	81	1	81	0.055	0.8172
FB	12.25	1	12.25	8.39 E-003	0.9283
AB	552.25	1	552.25	0.38	0.5484
D^2^	9865.95	1	9865.95	6.76	0.210
F^2^	6.57 E+005	1	6.57 E+005	449.63	˂0.0001
A^2^	6590.37	1	6590.37	4.51	0.0519
B^2^	5644.86	1	5644.86	3.87	0.0694
Residual	20440.08	14	1460.01		
Lack of Fit	16674.08	10	1667.41	1.77	0.3060
Pure Error	3766	4	941.50		
Cor Total	7.62 E+006	28			

**Table 8:** ANOVA for response surface quadratic model (Box-Behnken for yeast biomass response).

**Source**	**Sum of squares**	**df**	**Mean Square**	***f*** **-value**	***p*** **-value** **(Prob ˃ F)**
Model	39.43	14	2.82	31.41	˂0.0001
D- Glucose	14.74	1	14.74	164.38	˂0.0001
F- Aeration	21.28	1	21.28	237.30	˂0.0001
A- Selenium	0.072	1	0.072	0.80	0.3851
B- Temperature	0.22	1	0.22	2.41	0.1430
DF	1.60 E-003	1	1.60 E-003	0.018	0.8956
DA	0.20	1	0.20	2.21	0.1594
DB	0.26	1	0.26	2.84	0.1139
FA	9.03 E-003	1	9.03 E-003	0.10	0.7557
FB	2.03 E-003	1	2.03 E-003	0.023	0.8827
AB	0.011	1	0.011	0.12	0.7311
D^2^	2.62	1	2.62	29.22	˂0.0001
F^2^	5.03	1	5.03	8.34	˂0.0001
A^2^	0.071	1	0.071	0.79	0.3899
B^2^	0.055	1	0.055	0.61	0.4478
Residual	1.26	14	0.090		
Lack of Fit	1.18	10	0.12	6.70	0.059
Pure Error	0.071	4	0.018		
Cor Total	40.68	28			

**Figure 1:**
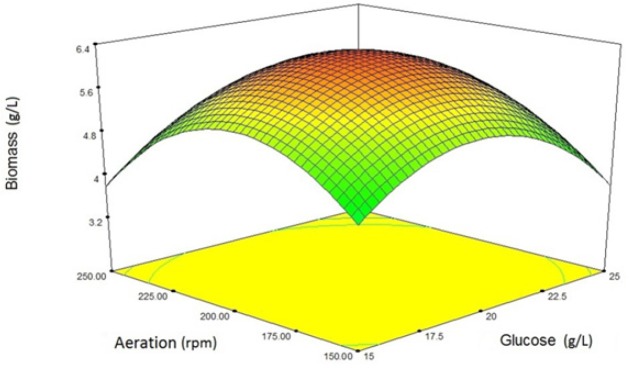
Three-dimensional diagram of the interaction of variables (glucose concentration and aeration) affecting the production of biomass

**Figure 2:**
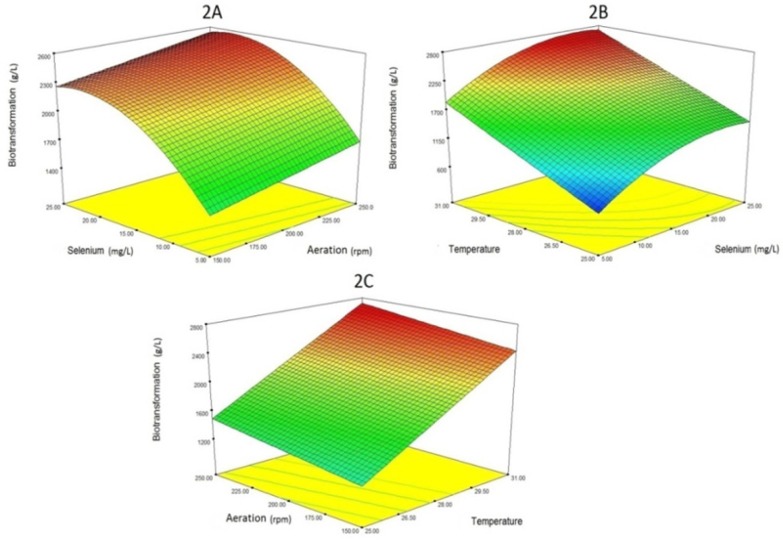
Three-dimensional diagram of the interaction of variables (selenium concentration, aeration and temperature) affecting the selenium biotransformation.

**Figure 3:**
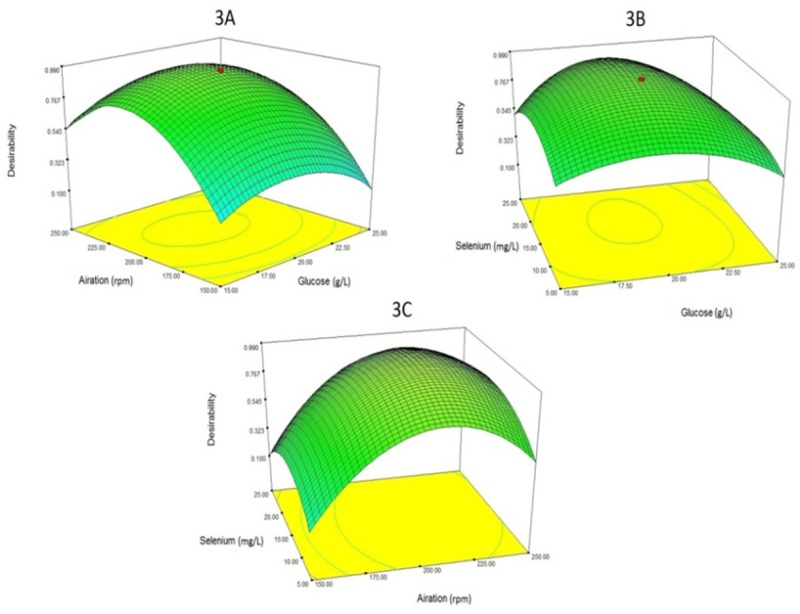
The relationship between "aeration", "glucose concentration" and "selenium concentration" with desirability (the desired level of both responses simultaneously(.

*Experimental design and statistical analysis for optimization*

Optimization of Seleno-yeast production by *Saccharomyces cerevisiae *isolate was done in two stages. First, the components that had important effect on Se biotransformation and biomass production were identified. Second, the optimum amount of these components was determined for Se biotransformation and biomass production (16). 

*Screening design*

Screening of significant components affecting Se biotransformation and biomass production by *Saccharomyces cerevisiae (S. cerevisiae)* was carried out by Plackett-Burman design separately. According to this design, eleven parameters were chosen for this study. For studying the effect, each of these components were represented at two levels (Table 1). Assuming that the variables do not interact with each other and a first-order multiple regression models is appropriate the following can be obtained:

Equation (1): $Y=\beta_{0}+\sum_{i=1}^{k}\left(\beta_{i}x_{i}\right)$

In Equation (1), Y is the response function (Se biotransformation or biomass production) and βi is the regression coefficient. The design matrix provided by the Design-Expert^®^ 7.0.0 software for the assessment of eleven parameters in twelve experimental trials is presented in Table 2. It should be noted that a separate table was designed for each response. With the aim of detecting the curvature, five central points of all parameters were also used in five separate batches (trials 13 to 17). According to Table 2, culture media were made and yeast cultivation was performed. At the end of the process, the dry weight of the biomass was measured and the amount of selenium biotransformation was determined in accordance with paragraph 2.2. The major effect of each parameter was obtained in accordance with Equation 2.

Equation (2): $E\left(\mathrm{xi}\right)=\frac{2(\sum \mathrm{Pi}+\sum \mathrm{Pi}-)}{N}$

In Equation (2), E (xi) is the effect of tested parameter, Pi + and Pi – indicate the response (Se biotransformation or biomass production) of the experiments where the tested parameter was introduced at high and low levels, respectively. Also N is the number of experimental trials (16). Data were analyzed by ANOVA (analysis of variance) and statistical significance was at *p*-value < 0.05.

*Optimization design*

After choosing the most significant parameters influencing Seleno-yeast production by *S. cerevisiae*, optimization of parameters (glucose concentration, aeration, selenium concentration and temperature) was done according to Box–Behnken design (16). The optimization of these parameters was to simultaneously optimize the biomass and selenium biotransformation. Selected parameters were studied at three levels. The actual and coded values of the parameters are given in Table 3. 

The design consisted of 29 reactions including 24 factorial points and 5 repetitions at the center point. Also the response values were Se biotransformation and yeast biomass production (Table 4). 

All experimental trials were repeated three times. Se biotransformation and biomass production were predicted by following second order polynomial Equation:

Equation (3): $Y=\beta_{0}+\beta_{1}A+\beta_{2}B+\beta_{3}D+\beta_{4}F+\beta_{11}A^{2}+\beta_{22}B^{2}+\beta_{33}D^{2}+\beta_{44}F^{2}+\beta_{12}\mathrm{AB}+\beta_{13}\mathrm{AD}+\beta_{14}\mathrm{AF}+\beta_{23}\mathrm{BD}+\beta_{24}\mathrm{BF}+\beta_{34}\mathrm{DF}$

Where Y is the response, β0 is the intercept (constant) and β_1_, β_2_, β_3_, and β_4_ are linear. Also β_11_, β_22_, β_33_ and β_44_ are squared coefficients and in addition, β_12_, β_13_, β_14_, β_23_, β_24_ and β_34_ are interaction coefficients. Finally, A, B, D, and F are the levels of, selenium concentration, temperature, glucose concentration and aeration respectively. Evaluation of the linear, quadratic and interactive effects of the independent parameters on the response is possible by means of this equation. Assessment of the effect of parameters on the response was performed by using ANOVA through Fisher’s test and the significances of all terms were judged by a *p*-value of < 0.05. For evaluation of the fitness of the second order polynomial equation, multiple correlation coefficients (R2) and adjusted R2 were applied. Ternary plot and contour plots were used to examine the interaction and relations between the parameters and the response. The optimum points were found by resolving the equation resulting from the final quadratic model and investigating in RSM diagrams.

## Result and Discussion

*The amount of biomass production and the organic selenium (selenium biotransformation) in the selected yeast strain*

Yeast (*S. cerevisiae*) isolate used in the work was obtained in our previous study. The selected strain was estimated for organic selenium and biomass production. After 24 h of incubation, organic selenium and biomass content were obtained 1855 ppm and 3.73 g/L respectively. 

*Screening of important medium components*

In order to determine the effects of independent factors on Se biotransformation and yeast biomass production, Plackett-Burman screening experimental design was used. Among the factors studied, selenium concentration (A), temperature (B), glucose concentration (D) and incubation time (J) were significant in Se biotransformation (Table 5). Also, glucose concentration (D), aeration (F), temperature (B) and selenium concentration (A) were significant in yeast biomass production (Table 6). As shown in Tables 5 and 6, both models are highly significant for both responses. Also, the model›s lack of fit is not significant in both models. This indicates that the models fit the data. Equations were derived from Plackett-Burman design were as follows:

Equation (4): Y1 = -1842.75 + 67.65 (A) + 128.83 (B) - 35.13 (D) - 6.47 (J)

Where Y1 is the response (organic Se or Se biotransformation), and A, B, D, and J are Se concentration, temperature, glucose concentration and incubation time, respectively.

Equation (5): Y2 = -1.41 + 0.31 (D) + 0.017 (F) - 0.13 (B) - 0.035 (A)

Where Y2 is the response (biomass production), and D, F, B, and A are glucose concentration, aeration, temperature and selenium concentration respectively.

As can be seen from Equation 4, Se concentration and temperature have positive effect, while glucose concentration and time fermentation had negative effect on Se biotransformation by *S. cerevisiae*. Also it can be seen from Equation 5 that glucose concentration and aeration exerted positive effect, whereas temperature and selenium concentration had negative effect on yeast biomass production. Nevertheless, the highly significant effect of curvature in both models means that at least one parameter is involved in an order higher than one. This suggests that a linear model is not suitable for determining the optimal Se biotransformation and yeast biomass production. As a result, a higher order model should be used. For this reason, simultaneous optimization of both responses was performed by Box-Behnken design. Additional statistical analysis showed that the difference among the means of factorial trials and center point in this study were not significant (*p* > 0.05). This demonstrated that the optimal levels for both of response are selected correctly and there was no need to employ the steepest ascend method (15, 16).

*Optimization of effective parameters with the aim of simultaneously optimizing the biomass and Se biotransformation*

In order to optimize both Se biotransformation and yeast biomass production at the same time, the factors that influence both responses should be selected and optimized. As is clear, glucose concentration, Se concentration and temperature affect both responses. Also, the aeration was selected as the fourth variable due to its significant effect on yeast strains metabolism. Accordingly, four effective factors (glucose concentration, aeration, Se concentration and temperature) were optimized using Box-Behnken response surface methodology, and acquired results were analyzed by ANOVA (Tables 7 and 8) (15). In the optimization section, glucose concentration, aeration, Se concentration and temperature parameters are marked with letters D, F, A and B respectively. As shown in Table 7, three linear (aeration, Se concentration and temperature) and one quadratic (aeration) terms were significant (*p*-value < 0.05). The model explaining the relationship between parameters (F, A, and B) and Se biotransformation (Y3) could be reduced to:

Equation (6): Y3 = -5745 + 2.77 (F) + 134.01 (A) + 204.05 (B) - 3.04 (F^2^)

Where Y3 is the response (Se biotransformation), and F, A, and B are aeration, Se concentration and temperature respectively. The positive coefficients of F, A and B indicate that Se biotransformation is increased by rising levels of aeration, Se concentration and temperature. The calculated value for the squared correlation coefficient (R2) and adjusted R2 are 0.97 and 0.93, respectively. This indicates that this model can illustrate 97% variability in the response and only less than 3% of the variability is caused by noise. Furthermore, the likeness between R2 and adjusted R2-values demonstrates the sufficiency of the model to predict the response. Also, The amount of the coefficient of variation (CV% = 6.71) shows the reliability and precision of the model.

Also in Table 8, which is related to the yeast biomass production; two linear (glucose concentration and aeration) and two quadratic (glucose concentration and aeration) terms were significant (*p*-value < 0.05). The model explaining the relationship between parameters (D and F) and yeast biomass production (Y4) could be reduced to:

Equation (7): Y4 = -15.08 + 1.18 (D) + 0.02 (F) - 0.02 (D^2^) - 0.01 (F^2^)

Where Y4 is the response (yeast biomass production), and D and F are glucose concentration and aeration respectively. The positive coefficients of D and F indicate that yeast biomass production increases by rising levels of glucose concentration and aeration. The calculated value for the squared correlation coefficient (R2) and adjusted R2 are 0.98 and 0.94, respectively. This indicates that this model can illustrate 98% variability in the response and only less than 2% of the variability is caused by noise. Furthermore, the likeness between R2 and adjusted R2-values demonstrates the sufficiency of the model to predict the response. Also, The amount of the coefficient of variation 

(CV% = 6.41) shows the reliability and precision of the model. In addition by omitting the insignificant factors in Table 7, the predicted R2 increased from 0.82 to 0.93. Also, in the case of Table 8, the predicted R2 increased from 0.80 to 0.94.

In the case of yeast biomass production, the response surface from the interaction between glucose concentration and aeration is illustrated in Figure 1. As it is clear from the Figure 1, increasing glucose concentration up to 20 g/L has a positive effect on the biomass production. But a further increase has a negative effect on the growth rate. This may be due to the Crabtree phenomenon. In the case of aeration rate, this relationship is also established. Excessive aeration may have adverse effects on yeast growth.

In the case of Se biotransformation (organic Se production), the response surface from the interaction between aeration, Se concentration, and temperature is illustrated in Figure 2. About the relationship between aeration and Se biotransformation, increasing aeration will partly increase Se biotransformation. About the effect of temperature on the Se biotransformation process, a direct relationship is observed. In fact, as the temperature rises, the amount of selenium biotransformation increases. Regarding the effect of selenium levels, it is also natural to increase the amount of organic selenium (Se biotransformation) by increasing the concentration of selenium. Of course, as is clear from the Figure 2, excessive selenium concentration (more than 20 mg/L) has no effect on the absorption of selenium. This phenomenon is probably due to the saturation of the selenium input channels.

According to the results, three factors of glucose concentration, Se concentration and temperature affect both responses. It can be optimized with these three factors, but if the factors increase, we can get better results in optimization. On the other hand, the use of five factors greatly increases the number of reactions. For this reason, four factors were used to optimization. “Aeration” was chosen as the fourth factor between aeration and fermentation time. The reason for this choice is that, according to the results, aeration factor has the second-highest impact on the biomass, while the fermentation time is fourth in the impact on Se biotransformation.

Finally, to maximize both responses (Se biotransformation and yeast biomass production), four factors of glucose concentration, aeration, Se concentration and temperature were optimized. The optimum levels for glucose concentration, aeration, Se concentration and temperature were determined to be 19.38 g/L, 213 rpm, 15.51 mg/L and 26 °C respectively. At these concentrations, predicted Se biotransformation (organic Se) and yeast biomass yield were calculated to be 2625 ppm and 5.98 g/L respectively. Figure 3 also shows 3 three-dimensional plots of the relationship of parameters with desirability (the optimum level of both responses simultaneously). In fact, Figure 3 is an optimized amount for both responses (Se biotransformation and yeast biomass yield).

*Verification of the optimal point predicted by the software*

For this purpose, cultivation of yeast strain was carried out according to the predicted points. The biomass and Se biotransformation were measured. The biomass and Se biotransformation were obtained 5.89 g/L and 2589 ppm, respectively (the results are repeated three times). Comparison of these values with predictive point was done by SPSS software. The results showed no significant difference between the two groups (results are not shown). Also, comparing the results of this study with previous studies shows that the amount of organic selenium produced by yeast is satisfactory to previous work. For example, Khosravi *et al.* were able to produce selenium-enriched yeast with 2690 ppm of organic selenium. But, it should be noted in that study, yeast biomass production had not been optimized. As a result, we can say that the overall results of our study are in a good level.

## Conclusion

In this investigation, the Plackett-Burman design was used to screen various factors and determination of factors affecting Se biotransformation and biomass production by *S. cerevisiae*. The screening was done for both responses separately. There are numerous reports describing the employ of this technique in medium optimization with a number of microorganisms (15, 16). According to Plackett-Burman design results it was found that glucose concentration, aeration, temperature and selenium concentration exhibit statistically significant effect on yeast biomass production. Also, it was found that 4 factors of selenium concentration, temperature, glucose concentration and fermentation time also had a significant effect on the Se biotransformation. In the next step, glucose concentration, aeration, Se concentration and temperature, which showed significant effect on Se biotransformation and biomass production in yeast, were chosen for optimization by means of Box-Behnken response surface methodology. In this research, simultaneous optimization of two responses (Se biotransformation and biomass production) was performed. This technique has been used in similar studies including optimization of culture parameters for selenium-enriched yeast production (11). By means of statistical experimental design, we could increase the amount of Se biotransformation in the yeast from 1855 ppm to 2589 ppm. In this way, the production of biomass in Seleno-yeast was also increased from 3.73 g/L to 5.89 g/L. The proximity of the predicted values to the values obtained from empirical experiments demonstrates the accuracy and feasibility of experimental design methods for the optimization of biological processes.
